# An Integrative Model for Understanding Obsessive-Compulsive Disorder: Merging Cognitive Behavioral Theory with Insights from Clinical Neuroscience

**DOI:** 10.3390/jcm11247379

**Published:** 2022-12-12

**Authors:** Eyal Kalanthroff, Michael G. Wheaton

**Affiliations:** 1 Department of Psychology, The Hebrew University of Jerusalem and Israel, Jerusalem 91905, Israel; 2 Department of Psychology, Barnard College, New York, NY 10027, USA

**Keywords:** obsessive-compulsive disorder (OCD), cognitive behavioral therapy (CBT), exposure and response prevention (EX/RP), executive control, habit, inhibition

## Abstract

Several models have been proposed for the emergence and maintenance of obsessive-compulsive disorder (OCD). Although these models have provided important insights and inspired treatment development, no single model has yet sufficiently accounted for the complexed phenotype of the disorder. In the current paper, we propose a novel model that integrates elements from cognitive behavioral models of OCD with neurocognitive approaches to the disorder. This Reciprocal Interaction Model (RIM) for OCD is based on two assumptions: (a) similar observed symptoms can stem from different etiological processes; and (b) neuropsychological deficits (such as reduced response inhibition and overreliance on the habit formation system) and cognitive behavioral processes (such as temporary reduction in anxiety after engaging in compulsive behaviors) mutually affect each other such that abnormalities in one system influence the second system and vice-versa—creating a vicious cycle of pathological processes. Indeed, the bidirectional inhibitory connection between anxiety/obsessions and executive control is at the heart of the model. We begin by briefly reviewing the current models for OCD. We then move on to describe the RIM, the supporting evidence for the model, the model’s predictions, and potential clinical implications.

## 1. Introduction

Obsessive-compulsive disorder (OCD) is a neuropsychiatric condition characterized by obsessions (intrusive and unwanted thoughts, images, or urges) and compulsions (repetitive behaviors and/or mental acts [1]). OCD is estimated to affect up to 2% of the population [2], and its symptoms can be disabling when severe [3,4]. As such, OCD has been recognized as a significant global cause of non-fatal illness burden [5]. OCD patients present with a range of insight; although some are convinced to a delusional degree, most patients recognize that their concerns are excessive and irrational [1]. Why these individuals are not able stop engaging in their symptoms has been the focus of substantial research across divergent disciplines including clinical psychology, psychiatry, and cognitive neuroscience. Across these diverse disciplines, researchers have developed parallel models to account for the development and maintenance of OCD. In one vein of inquiry, clinical psychologists developed and refined cognitive behavioral models of OCD in relation to developing effective forms of psychotherapeutic treatment. In parallel, cognitive neuroscientists have modeled the neurocognitive mechanisms of OCD with a particular emphasis on domains of executive control. For the most part, however, these models have not been fully integrated or reconciled. Thus, an integrative model is warranted, which we set out to accomplish in the present paper. We begin by describing some of the prevailing OCD models and their limitations. Next, we propose a novel integrative model for OCD and review the literature that is consistent with it. Finally, we conclude with a series of predictions by which future studies could empirically test and refine this model. We also address clinical implications for how treatments could be improved based on the model.

### 1.1. Cognitive Behavioral Models of OCD

Dollard and Miller [6] are often credited with articulating the earliest behavioral conceptual model of the development and maintenance of OCD symptoms by adapting Mowrer’s two-stage theory [7,8]. According to this model, fears are acquired and generalized through classical conditioning and maintained through avoidance and escape behaviors (which are negatively reinforced). The key to this model is the functional connection between obsessions and compulsions: obsessions provoke distress (anxiety, disgust, and discomfort), which is subsequently reduced by compulsions [9]. This functional connection between obsessions and compulsions has been demonstrated experimentally [10,11]: the short-term relief provided by compulsions negatively reinforces the behavior, which results in the behavioral tendency for compulsions to increase over time. At the same time, engaging in compulsions prevents the habituation of the distress associated with obsessions [12]. Thus, compulsive behaviors that OCD patients typically perform may lead to short-term relief but paradoxically increase obsessional anxiety over time, effectively perpetuating compulsions [13]. Focusing on this connection between obsessions and compulsions, behavioral therapists developed *exposure and ritual prevention* (EX/RP) [14,15] treatment as a way to help patients break free from the cycle. Treatment involves exposing the patient to the very events that evoke obsessional distress while refraining from compulsive rituals. This treatment is the most thoroughly empirically supported treatment for OCD and is currently recommended as a first line OCD treatment [16,17,18].

**Figure 1:**
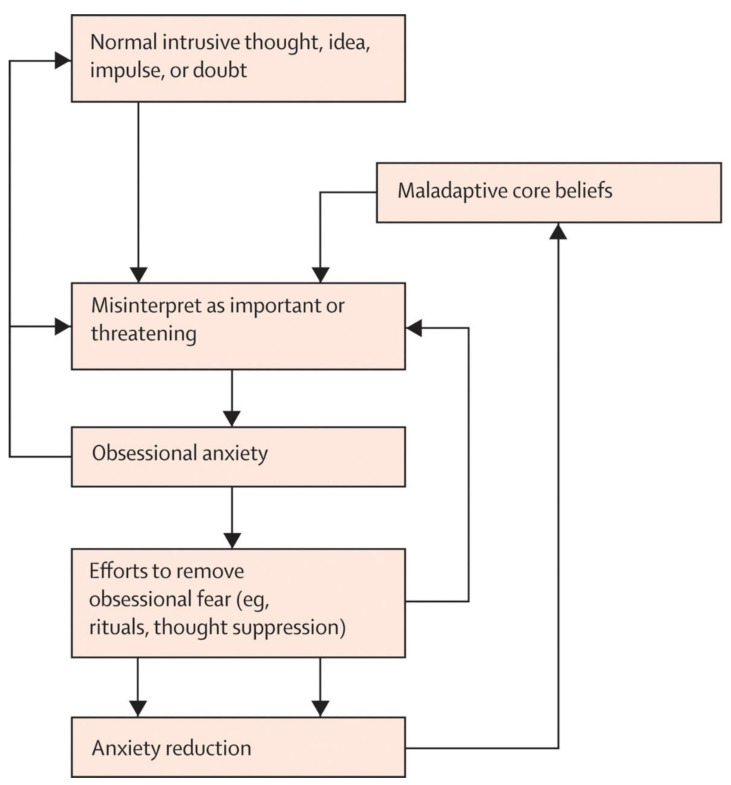
Cognitive behavioral model of OCD (reproduced with permission from Abramowitz, 2009 [9].

The behavioral model of OCD was subsequently expanded to account for the important role that beliefs and appraisals play in the disorder. It is a well-established finding that unwanted cognitive intrusions (i.e., unpleasant thoughts, images, and urges) with contents similar to clinical obsessions are experienced by most people in the general population [19] and thus intrusive thoughts are not in themselves pathological per se [20]. Appraisal-based models suggest that intrusive thoughts are elevated to clinical obsessions when they are misinterpreted as being significant or dangerous [21]. From this perspective, cognitive models of OCD explored particular domains of beliefs that could contribute to this cycle (termed *obsessive beliefs*), including beliefs about responsibility [22], beliefs that thoughts are equivalent to or lead to actions (referred to as *thought–action fusion* [23]), and meta-cognitions about the meaning and consequences of thoughts [24]. Cognitive therapy for OCD, which has also been supported by research [25,26], involves directly targeting and modifying these maladaptive beliefs and misinterpretations. Contemporary cognitive behavioral models of OCD involve a synthesis of the behavioral and cognitive elements described above (Abramowitz and Jacoby, 2015) and as illustrated in Figure 1.

Although CBT models have received empirical support and led to effective treatments, they are not without drawbacks, as evident in the fact that not all patients equally benefit from CBT. Across multiple studies, 25–35% discontinue EX/RP prematurely and up to 20% who complete treatment do not experience a treatment response [27]. Moreover, these models do not incorporate research from the field of cognitive neuroscience to account for OCD’s unique neurocognitive profile.

### 1.2. Neurocognitive Models of OCD

Neurocognitive models of OCD have focused on executive control as a causal mechanism that underlies OCD symptoms. Executive control is a key human function that allows us to guide behavior in accordance with our internal goals by governing our efforts to achieve and maintain goal-directed behavior. It has been suggested that executive control can be divided into three main components: inhibition, working memory, and task-shifting/flexibility [28,29,30]. Some accounts of OCD suggest that a deficit in key elements of the executive control system underlies the disorder’s clinical symptoms. Consistent with this view, there is evidence suggesting that OCD patients demonstrate neuropsychological deficits in executive control tasks [31,32,33,34]. In one meta-analysis (of 3162 individuals with OCD and 3153 healthy control participants across 84 studies), Snyder and collogues [35] found that OCD was characterized by a “broad spectrum” deficit to the executive control system. This conclusion was also supported by neuroimaging findings from the brain. Although a full review of the imaging literature is beyond the scope of the present article, studies show that that patients with OCD display abnormalities relative to controls in the cortico-striato-thalamic-cortical (CSTC) circuitry [36,37], including volumetric gray-matter reductions and reduced white-matter integrity in the anterior cingulate cortex [38], gray-matter reductions in the orbitofrontal cortex [39], and gray-matter increases in the thalamus and ventral striatum [40]. The CSTC loop circuits—specifically the prefrontal areas that are a part of these circuits—are important for self-regulation of affect, cognition, and behavior [41,42] and thus play a central role in the broad spectrum of executive functions. Although performance deficits have been noted in many executive control tasks, the most fully articulated neurocognitive models of OCD have focused on two specific domains of executive control: response inhibition and habit-based systems. Although other functions (e.g., set shifting, planning, and working memory) have also been implicated in OCD, we focused on these two given that these functions have generally had less-consistent findings in meta analyses [42,43].

**Figure 2:**
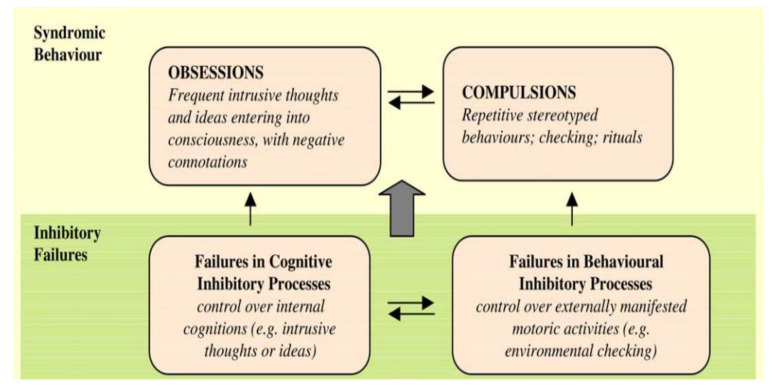
Model of OCD focused on inhibitory failure (reproduced with permission from Chamberlain et al., 2005 [44]).

Perhaps the most prominent executive control model of OCD focused on inhibitory control—the ability to suppress irrelevant thoughts or actions [30]. Chamberlain and collogues [44] developed a model that posits that dysfunctional inhibitory control underlies both obsessions and compulsions (see Figure 2). That is, both the repetitive thoughts and behaviors should be inhibited by the executive control system but are not because of a deficit in the system. In turn, these uninhibited intrusions and/behaviors reinforce each other. Importantly, according to this model, an inhibitory deficit represents an underlying causal factor for the onset and maintenance of the disorder that is presumed to exist prior to OCD onset [29,30,31,32,33,34,35,44]. In line with this model, several investigations reported worse performance on tasks that required response inhibition in OCD patients [45,46,47,48,49]. One study reported a similar result in unaffected first-degree family members of OCD patients [50]. Based on these findings, some researchers have suggested that deficient response inhibition is the mechanism that underlies repetitive thoughts and behaviors in OCD. Thus, deficits in response inhibition have been suggested to be an endophenotype of OCD [47,50,51] see also [52,53].

A more recent neurocognitive model of OCD that emphasizes the role of the executive control system focuses on the (im)balance between “goal directed” and habit systems. This model posits that “compulsivity reflects the aberrant dysregulation of stimulus-response habit learning” [54] (p. 83), meaning that compulsions result from an excessive bias toward habitual responses [55,56,57], which underlies the imbalance between habit and goal-directed behavior. This model includes the rather provocative possibility that obsessions may develop secondary to compulsions as post hoc rationalizations of otherwise inexplicable (habitual) compulsive behaviors [58]. This hypothesis stems from several experimental studies that used response–outcome information-updating tasks and found that patients with OCD were more prone to “slips of action”—responding to a devalued stimulus (i.e., a stimulus that was no longer rewarded) whereas healthy controls (HCs) were more efficient in updating response–outcome information [59]. A recent work that administered a two-stage learning task to a sample of OCD patients before and after treatment with EX/RP found that that goal-directed planning was not affected by treatment, which suggested that deficits in goal-directed planning may be a stable vulnerability factor for OCD [60].

As can be seen, these executive control models have spurred useful debate and stimulated new ways of thinking about OCD. However, these neurocognitive models are not without drawbacks and limitations. Abramovitch, Abramowitz, and Mittelman [61] concluded their recent meta-analysis by suggesting that an executive control deficit in OCD is very small and fragile—if it exists at all—and is most likely “clinically insignificant”. These researchers further proposed that the deficit could represent a so-called epiphenomenon of OCD symptoms (i.e., an illness consequence rather than a cause). Indeed, studies of response inhibition in OCD yielded modest effect sizes and inconsistent results [44,62,63]. Furthermore, several studies have reported that the magnitude of the deficit in response inhibition was not correlated with OCD symptom severity [50,64,65,66] nor specific to OCD [38,54,57,58,67].

In a recent paper, Kalanthroff et al. [68] reviewed the evidence supporting the habit-formation model but also raised critical issues that this model does not adequately deal with. For example, this review paper emphasized that to date there is no strong evidence that the imbalance between the goal-directed and the habit-formation systems is the cause rather than the result of other OCD symptoms such as increased anxiety or the constant need to inhibit intrusive thoughts [69,70]. In addition, this paper pointed out that we currently do not have strong evidence to support the notion that compulsions precede the development of obsessions. Finally, a bias toward habits was also documented in other disorders such as social anxiety [71,72]; a recent paper emphasized that the imbalance between the executive control and habit system exists in several psychopathologies [55]. Taken together, it seems clear that deficient executive control is relevant for many individuals with OCD. However, the clinical phenotype cannot be entirely mapped onto the models that focus on executive control deficits. These models do not sufficiently explain why impairment in the executive system does not always lead to OCD and how individuals with OCD are often able to easily maintain goal-directed behavior in many fields of their lives.

## 2. Reciprocal Interaction Model (RIM) for OCD

As reviewed above, the existing models of OCD explain much of the phenotype of the disorder and have led to effective treatments in the form of CBT. In the current paper, we sought to develop a model that integrates and extends previous models in order to account for a greater part of the heterogeneity of the disorder.

### 2.1. Model Assumptions

**Figure 3:**
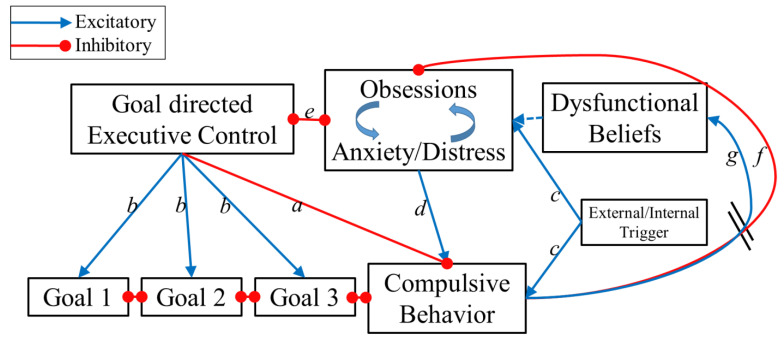
The Reciprocal Interaction Model (RIM) for OCD. Note that some connections are inhibitory (red) while some are excitatory (blue); in addition, some connections are unidirectional and some are bidirectional. The different connections are marked by letters and explained in the text.

The Reciprocal Interaction Model (RIM) for OCD aims to account for the large heterogeneity in the disorder’s symptoms while remaining parsimonious. The RIM adapts the basic structure of the prevailing cognitive behavioral model of OCD [73] to include the role of executive control. The RIM of OCD is presented in Figure 3. The model is based on the following key assumptions:

1. Similar to all previous OCD models, the RIM includes a self-maintained “vicious cycle” of obsessions and compulsions based on the functional relationship between the two: compulsions temporarily reduce distress associated with obsessions, thereby leading to more compulsions in the long term through negative reinforcement [9].
2. The model suggests more than one “entrance point” to the cycle that may vary between patients. In this way, the model embraces the idea of equifinality in that OCD patients share a similar endpoint (OCD status) even though they may have different etiologies and symptoms. The latter is consistent with the Research Domain Criteria (RDoC) approach, which suggests that even when the manifest form of symptoms might be similar (e.g., excessive handwashing), the processes underlying these symptoms might vary from one individual to another.
3. The model incorporates elements from previous appraisal-based models of OCD [21] reflecting that dysfunctional beliefs (or cognitive biases) contribute to the development of obsessions for some individuals.
4. Finally, in line with the ego-dystonic nature of OCD, this model proposes that most compulsive behaviors are not in line with the patients’ values and goals (represented as connections in Figure 1). Therefore, engaging in compulsions indicates that executive control has failed to inhibit compulsions. Problems in the executive control system may contribute to OCD vulnerability for some (but not all) patients.

### 2.2. Description of the Model’s Elements and Pathways

The core of this model is a bidirectional connection between obsessions/anxiety and goal-directed behavior (connection e in Figure 3). Executive control aims to achieve and maintain goal-directed behavior by inhibiting disturbing and interfering compulsive behaviors (connection a) while prioritizing other relevant goal-directed tasks (b). Furthermore, as shown in the recent literature, the executive control system has the ability to attenuate anxiety [74,75], distress [76], and intrusive thoughts [52,77,78,79]. Thus, when this unit is adequately activated, compulsions are significantly less likely to occur and obsessions and anxiety/distress are significantly reduced. However, this control unit is subjected to interference from the obsessions and the anxiety/distress units (connection e). When these units are sufficiently strong, they impair the executive control unit’s ability to resist the compulsive urges. Thus, when anxiety/distress is low and executive control is high, OCD symptoms are not likely to occur. However, if either anxiety/distress is high or executive control is low, the individual is at risk to develop OCD and the interplay between executive control and anxiety/distress will determine the course of the disorder. If the anxiety/distress develops into recurrent obsessions, then a vicious loop of more anxiety and less executive control will emerge. Subsequently obsessions increase compulsive urges (connection d). Once the compulsive behavior unit reaches a certain threshold, a compulsion is executed. Thus the two main innovations of the RIM are: (a) compulsions are a result of several possible interactions between executive control and obsessions and anxiety/distress; and (b) the competition between executive control and obsessions + anxiety/distress is the core of the disorder phenotype.

Connections in the model exist such that compulsions can be activated without obsessions/anxiety, which is in line with habit-based findings above [54] as well as clinical observations that some patients sometimes engage in compulsions outside of awareness (routinized compulsions done “automatically” in response to environmental cues or in order to pre-empt the activation of anxiety). Specifically, following the habit-formation and stimulus-driven behavior models, the RIM suggests that the compulsive urge can be directly triggered by external stimuli (connection c). Importantly, this does not suggest that all compulsive behaviors necessarily begin as stimulus-driven behavior; rather, some compulsions might be stimulus-driven, having developed into habits following years of repeating OCD behaviors. Connections also exist in the RIM such that compulsions can be activated without a pre-existing executive deficit; specifically, if obsessions and anxiety/distress are high enough and the compulsive urge is triggered (connection c). Thus, even an intact executive control system might fail in inhibiting compulsions under certain conditions. Importantly, we adopted the CBT models’ suggestion that once executed, compulsions provide a temporary reduction in obsessions and distress/anxiety (connection f). When anxiety/distress has been sufficiently reduced, the individual will be able to shift from compulsive behavior back into goal-directed actions. However, if anxiety/distress remains sufficiently strong, the cycle will continue for multiple rounds. The flexibility inherent in this model allows for situational variation in patients’ symptom severity in line with clinical observations: on some “good days” patients might report that 5 min of handwashing feels like enough, while on “bad days” (due to heightened anxiety or other factors) an increasing number of compulsions (e.g., 15 min of handwashing) may be required before the cycle is complete. This proposed element is in line with findings that OCD rituals increase in severity during times of general stress (e.g., university students during final exam week [80]). Each repetition of the cycle reinforces the long-term “dysfunctional belief” and strengthens the action tendency for future compulsions (connection g). Moreover, such flexibility is also consistent with clinical observations that indicate intact executive control in “non-OCD” circumstances (i.e., when obsessions and anxiety/distress are low).

As can be seen, the RIM accounts for the fact OCD is heterogeneous and can involve a complex interaction between contributing factors. For some people, a pre-existing deficit in executive control may make them less able to maintain goal-directed behaviors and more likely to give in to compulsions. For some patients, compulsions might start as habitual stimulus-driven behaviors. For others, anxiety and distress may be particularly strong and cause interference in the executive system that then leads to compulsions. Of course, different interactions between all these factors might characterize different patients. It should also be noted that the pattern becomes self-sustaining over time due to the negative reinforcement that compulsions yield. This heterogeneity even extends to intra-person variability in behavior depending upon situational factors.

### 2.3. Supporting Evidence for the Model’s Elements and Pathways

In the current paper, we propose a model of OCD that integrates and elaborates on elements of prior models. Most of the different components of this model have already been demonstrated in the literature; the novel contribution of our work is the synthesis of existing models. Specifically, some of the connections in our model (i.e., *d*, *g*, and *f* in Figure 3) were borrowed from the cognitive behavioral models [73]. However, other connections of the RIM are supported by new evidence that came to light in recent years. In the Section 2.3.1, we will briefly review the supporting evidence.

#### 2.3.1. The Effect of Obsessions and Anxiety/Distress on Executive Control (Connection e in Figure 3)

The effect of anxiety on executive control is well documented in both healthy populations and in anxiety patients. One of the outstanding examples of this research is the attentional control theory [81,82,83]. According to this theory, anxiety impairs the efficiency of the central executive system, thereby biasing the attentional system toward bottom-up/stimulus-driven processing. This theory is supported by a great deal of empirical support [81], which indicates that trait anxiety impairs response inhibition in anti-saccade [84,85,86,87], flanker [88], Stroop [70], and stop-signal tasks [75]. In addition, the notion that anxiety has a detrimental effect on the executive control system also comes from studies that showed opposed neural responses in the dorsal-executive versus the ventral-emotional systems in response to emotional distractors in an executive control task [89,90,91,92]. For example, these studies by Dolcos and colleagues found that “increased activity in the ventral affective regions in the presence of emotional distracters, temporarily takes offline the dorsal executive system” [92] (p. 3).

The effect of obsessions on the executive system has not been investigated directly. However, recent work provides initial evidence for the potential connection between obsessions and executive control. Kalanthroff, Aslan, and Dar [60] found that a manipulation in healthy controls to induce a threatened morality (a common obsession in OCD) has a detrimental effect on performance in several executive tasks such as the Stroop and stop-signal tasks. Importantly, in this study anxiety was not controlled for and thus might have underlain the effect. Alternatively, Abramovitch et al. [93] proposed that the executive control impairments in OCD are a result of continuous attempts to control obsessive thoughts that overload the executive system. Importantly, in our model, obsessions can impair executive control either through the anxiety they generate or by overloading the cognitive system.

#### 2.3.2. The Effect of Executive Control on Obsessions and Anxiety/Distress (Connection *e* in Figure 3)

As mentioned above, connection *e* is bidirectional. Evidence for the ability of executive control to regulate anxiety/distress stems from studies that showed that priming and training executive control could reduce emotional-interference effects. In a series of studies conducted with healthy controls, it was demonstrated that executive control can reduce emotional interference effects. For example, Cohen, Henik, and Mor [74] found that the emotional-interference effect (i.e., longer reaction times to negative images) is diminished following conflict-laden trails that require the activation of the executive control system (on several executive control tasks [75]). Furthermore, these researchers found that training the executive system by using a computerized task (i.e., Flanker) led to reduced amygdala reactivity to aversive information [76]. Similarly, Sari and collogues [94] showed that executive control training could reduce anxiety. To the extent that compulsive behaviors are motivated by anxiety, these studies suggest that compulsions could be indirectly reduced to the extent that efficient executive control relieves anxiety.

There is also direct evidence for the effect of executive control on obsessions and compulsion-like symptoms. Linkovski and collogues [52] used a repeated checking manipulation that has been shown to increase memory distrust [95,96] and demonstrated that efficient response inhibition (as measured by the stop-signal task) might “protect” from memory distrust (and the urge to recheck), albeit in healthy controls. In another study [77] that utilized healthy control participants, researchers found that triggering inhibition (by employing a task that requires frequent stopping) reduced scanning in the visual search task—a known behavioral manifestation of uncertainty [97].

It is important to mention here that according the RIM, although impairment in the executive control system might predispose individuals to develop OCD, such a deficit is not always sufficient nor is it necessary to develop OCD symptoms. In other words, the RIM suggests that the existence of both anxiety and impaired executive control is most likely to be a significant vulnerability factor to develop OCD. This suggestion will be further discussed below.

#### 2.3.3. The Effect of Executive Control on Compulsive Behavior (Connection a in Figure 3)

The RIM suggests that executive control has the ability to suppress the compulsive behaviors in favor of goal-directed actions. This is consistent with a series of studies by Gillan et al. [59,98] that demonstrated that among OCD patients, reduced activation of the (executive) “goal directed” system is associated with more compulsive behavior. Similarly, in another study [99], researchers demonstrated that task control, which is an executive control mechanism that is responsible for adaptive task-selection processes while suppressing irrelevant automatic tasks, was impaired in individuals with OCD. Moreover, these researchers showed that the size of this deficit correlated with the severity of the OCD symptoms.

Additional support for the effect of executive control on compulsions comes from sleep and alertness studies. The relationship between the duration of sleep and OCD symptoms has been established in several previous studies that documented a correlation between a shorter sleep duration and OCD symptoms. In a recent study, Nota and colleagues [100] found evidence that a shorter sleep duration was associated with worse inhibitory performance on a go/no-go task but only in individuals with more severe OCD symptoms. These researchers concluded that a lack of proper sleep makes it harder to inhibit repetitive thought and behaviors and thus increases compulsive behaviors. Similarly, Kalanthroff et al. [101] proposed that alertness, not lack of sleep per se, may drive the impaired executive control and thus OCD symptoms. The latter was based on studies that showed a correlation between reduced alertness and impaired inhibition [102,103,104] and on studies that showed better control over compulsive behavior when alertness was higher [105,106]

#### 2.3.4. Triggers of Compulsive Behaviors (Connection c in Figure 3)

The RIM suggests that both external and internal cues can directly trigger compulsive urges. As mentioned above, some researchers have suggested that compulsions might be “stimulus-driven behaviors”. The notion of stimulus-driven behaviors is based on findings that showed that stimuli have the ability to evoke the performance of an associated task (e.g., seeing a sink evokes handwashing [107,108,109]). Monsell [110] proposed that task sets can be activated not only by deliberate intentions (“endogenous”) but also by the perception of a stimulus attribute that is strongly associated with a particular task set (“exogenous”). This notion has been supported by studies of “motor evoked potentials” that demonstrated that stimuli can trigger the motor-planning and motor-execution brain regions [111,112,113].

In OCD, the notion that compulsions might be “stimulus driven” harkens back to an early-childhood OCD model proposed by Rapoport, Swedo, and Leonard [114]. Stimulus-driven behaviors were linked to compulsions in adults with OCD in the recent experimental works by both Gillan et al. [59] and Kalanthroff et al. [99]. Another line of studies suggested that OCD patient exhibit increased action tendencies that increase the likelihood of stimulus-driven behaviors. An action tendency is defined as a change in action readiness that is elicited by events. Using event-related potentials, Dayan, Berger, and Anholt [115] found a higher “readiness potential” upon the appearance of a target stimulus, which indicated preparation for action, in participants with high versus low OCD traits as well as in patients who were diagnosed with OCD. Finally, the ‘seeking proxies for internal states’ (SPIS) model also suggests that individuals with OCD tend to rely more on external sources of information, due to attenuated access to internal states [116].

#### 2.3.5. The Relationship between Compulsions and Dysfunctional Beliefs

The notion that compulsions reinforce dysfunctional beliefs was illustrated in van den Hout′s seminal work with healthy subjects, which demonstrated that compulsive-like behaviors such as checking were enough to induce memory distrust in healthy subjects [95]. In addition, van Uijen and Toffolo [96] found that healthy participants who were asked to conduct repetitive checking behaviors for one week reported more cognitive intrusions compared to participants who were not asked to conduct repetitive checking. Finally, Robbins et al. [54] suggested that obsessions are post hoc *rationalizations* of otherwise inexplicable (habitual) behaviors. This suggestion harkens back to William James, who described over 100 years ago that people frequently gave post hoc explanations of their own behaviors [117,118]. Post hoc explanations have also been used in theories of moral reasoning and cognitive dissonance. Gillan and Sahakian reviewed evidence that some OCD patients generate post hoc rationalizations and concluded that they “…erroneously deduc[ed] that if they felt driven to perform an act of (habitual) avoidance, they must have had something to fear” [58] (p. 248; see also [119]). Other accounts suggested that compulsions do not necessarily give rise to maladaptive beliefs, but rather that compulsions may contribute to the *maintenance* of pre-existing maladaptive beliefs by preventing their disconfirmation [120].

#### 2.3.6. The Relationship between Compulsions and Obsessional Thoughts (Connection f in Figure 3)

Finally, the notion that compulsions reduce distress provoked by obsessional intrusive thoughts comes from cognitive behavioral models of OCD [10,11]. The distress-reducing quality of compulsive behaviors may extend beyond OCD patients to even healthy individuals: a recent work demonstrated that even in healthy participants, handwashing reduced negative emotional cognitive experiences. In a series of experiments, Zhong and Liljenquist [121] established a connection between bodily purity and moral purity. They found that participants who were asked to recall or write down an immoral deed were more likely to incorporate cleansing-related words in a word-completion task (Experiment 1), to express preferences for cleansing products (Experiment 2), and to choose a cleansing-related gift (Experiment 3). In addition, after being asked to recall an immoral deed, participants were more likely to volunteer for an additional study, presumably as a means to restore their sense of morality (Experiment 4). Crucially, this effect was reduced if participants were given a chance to wash their hands after recalling the immoral deed. The latter finding was found to be even more robust in OCD patients [122]

## 3. Discussion

The Reciprocal Interaction Model (RIM) for OCD integrates prevailing clinical and neurocognitive models and incorporates recent findings in the literature in both healthy control subjects and OCD patients to form an integrative model of OCD. The model assumes that different processes that interact with each other can lead to the complex phenotype of OCD. As demonstrated above, the model is consistent with previous findings in the literature. However, the model also makes some critical testable predications. In addition, the model might have some novel clinical implications, which are discussed below.

### 3.1. Model Predictions and Clinical Implications

#### 3.1.1. Subtypes of OCD

In line with the Research Domain Criteria (RDoC) initiative’s call to integrate many levels of information to further our understanding of basic transdiagnostic dimensions of functioning [123], the RIM suggests that data-driven clusters not based on symptoms per se can be formed based on two dimensions: anxiety levels and executive control efficiency. Specifically, some patients could be more “anxiety driven”, others could be more “executive control deficit driven”, and some might be driven by both high anxiety and deficient executive control. Thus, the model may be a step toward precision medicine. Understanding the patients’ unique cycle characteristics may provide specific treatment targets. For example, EX/RP is the gold-standard treatment for OCD. The treatment consists of exposure (to stimuli and situations that evoke compulsions) and response prevention [124,125]. Patient adherence to ritual prevention strongly predicts treatment outcome and thus is crucial to successful treatment [126]. It has been suggested that ritual prevention may be contingent upon executive control [127]. Thus, for patients with poor executive control it might be necessary to strengthen the executive control ability in order to allow successful EX/RP treatment. This could be done in multiple ways; for example: computerized cognitive training or adjunctive-alertness-based treatments (e.g., circadian rhythm, physical exercise, and light treatment [106]). As mentioned above and in line with the predictions of the RIM, it has been shown that executive control training can reduce anxiety [94]. This supports the notion that executive control training can support EX/RP. On the other hand, SSRIs, which are the first-line pharmacological treatment for OCD, do not directly affect the executive control system [128,129,130] and thus might be efficient only through reducing anxiety. Hence, SSRIs might prove beneficial for patients who are mainly “anxiety-driven” but who do not exhibit a significant executive control deficit. Interestingly, this suggestion might explain why SSRI pharmacotherapy is typically associated with only a partial response in OCD symptoms. It is important to note that other non-exposure-based treatments have been proven useful for OCD patient, such as Acceptance and Commitment Therapy (ACT), Interpersonal Therapy (IPT), and Mindfulness-Based Stress Reduction (MBSR).

#### 3.1.2. Reduced Executive Control Predicts Engagement in Compulsive Behaviors

As elaborated above, the core feature of the RIM is the bidirectional inhibitory connection between the executive control unit and obsessions/anxiety unit. The model predicts that activation of this subsystem of executive control and obsessions/anxiety will predict engagement in compulsive behaviors. Thus, in situations that negatively affect the executive control system—whether as result of increased anxiety or as a result of a different factor (e.g., low alertness)—OCD patients will be more likely to engage in compulsive behaviors. The bidirectional inhibitory connection suggests that a reduction in executive control will in turn increase anxiety—both because of the reduced suppression of the anxiety/obsessions system and because of the indirect amplification of the obsessions/anxiety system via the dysfunctional beliefs unit. If confirmed, this model-driven insight might shed light on day-to-day fluctuations in OCD symptom severity. For example, given the known connection between the arousal system and the executive control system [102,103,104], it might be suggested that in a low-arousal situation, patients might experience difficulty in resisting their compulsions [100,101,105,106]. Similarly, antecedents that weaken executive control (e.g., sleep deprivation) would be expected to be associated with more lengthy cycles of compulsions.

#### 3.1.3. Robust Executive Control Might Prevent Compulsions (and in Turn OCD)

The RIM suggests that executive control can suppress irrelevant thoughts (e.g., obsessions and anxiety) and behaviors (e.g., compulsions). As mentioned above, this notion was supported by a few recent studies [69,77]. Thus, although the model does not suggest that poor executive control *leads* to OCD (because OCD can also result from increased obsessions/anxiety), the model does suggest that robust executive control can make the development of OCD less likely. In other words, the model predicts that individuals with particularly efficient inhibitory control are much less likely to develop OCD. The model predicts that individuals with very good executive control might still develop OCD when overwhelming obsessions/anxiety are evident. Similarly, since the model predicts that a both high obsessions/anxiety and low executive control are risk factors for developing OCD, it is reasonable to also predict that having both poor executive control and/or high obsessions/anxiety will additively increase the risk for developing OCD.

#### 3.1.4. Executive Control Deficits Will Be Evident When OCD Symptoms Are Provoked

As mentioned above, the RIM does not imply that all OCD patients will necessarily exhibit deficient executive control across all situations (i.e., a trait-level deficit). However, given the reciprocal inhibitory connection between executive control and obsessions/anxiety, the model predicts that even if executive control is not globally impaired, under conditions involving heightened anxiety, executive performance may be temporarily reduced. Thus, a key prediction of this model is that in some cases, an executive control deficit in OCD might be state-dependent—obsessions/anxiety may cause a momentary disruption in executive control even among those patients who do not have a pre-existing trait-level deficit in executive control. The RIM explains why many OCD patients are able to achieve and maintain goal-directed behavior in their everyday lives (outside of the disorder) by suggesting that for those patients, executive deficits will be mainly evident when obsessions/anxiety are triggered. This possibility might also explain some of the mixed literature on neurocognitive deficits in OCD patient samples.

### 3.2. Summary

In the current paper, we proposed a novel Reciprocal Interaction Model of OCD that integrates and expands upon two prevailing models of OCD: the cognitive behavioral and neurocognitive conceptualizations. We reviewed the existing evidence that supports the different components of the model and suggested additional predictions that were driven by the model. To explain the complex phenotype of OCD, the RIM emphasizes the interplay between different components rather than focusing on single a factor as a causal mechanism (i.e., a single deficit as the specific mechanism). In this way, the RIM is in line with the drive toward precision medicine in OCD. Specifically, OCD symptoms are heterogeneous in presentation and may also be varied in the underlying cause. If clinicians were able to delve beneath overt symptoms to reveal specific contributing factors (i.e., differential pathways to OCD), they might be better able to individualize the treatment. Furthermore, we hope that the RIM will spur future research that integrates clinical psychology and cognitive neuroscience. For too long, these disciplines have tended to operate independently (though often in parallel). Instead, we hope they will work together and combine efforts to improve the understanding of OCD. Importantly, the model we proposed here did not include all potentially relevant contributing factors; future research might add additional variables to the model (e.g., disgust sensitivity). Finally, the RIM might also spur researchers and clinicians to develop similar models for other disorders that could also benefit from a more nuanced understanding of the underlying mechanisms.

## Data Availability

Not applicable.
